# Interleukin-34 Synovial Fluid Was Associated with Knee Osteoarthritis Severity: A Cross-Sectional Study in Knee Osteoarthritis Patients in Different Radiographic Stages

**DOI:** 10.1155/2018/2095480

**Published:** 2018-08-12

**Authors:** Shuang-Lei Wang, Rui Zhang, Kong-Zu Hu, Mao-Qiang Li, Zhan-Chun Li

**Affiliations:** ^1^Department of Orthopaedic Surgery, Renji Hospital, School of Medicine, Shanghai Jiaotong University, Shanghai, China; ^2^Department of Orthopaedic Surgery, The First Affiliated Hospital of Anhui Medical University, Anhui, China; ^3^Department of Orthopaedic Surgery, Hangzhou First People's Hospital, Zhejiang, China

## Abstract

**Background:**

Inflammation might play a crucial role in the pathogenesis of osteoarthritis (OA). Interleukin-34 (IL-34) is a well-known proinflammatory cytokine.

**Objective:**

The objective of this study was to detect IL-34 levels in serum and synovial fluid (SF) of patients with OA and to investigate their correlation with radiographic and symptomatic severity.

**Methods:**

One hundred and eighty-two OA patients and 69 controls were recruited. IL-34 levels were measured by enzyme-linked immunosorbent assay (ELISA). Radiographic and symptomatic severity of OA was reflected by Kellgren-Lawrence (KL) grades and Western Ontario McMaster University Osteoarthritis Index (WOMAC) scores, respectively.

**Results:**

SF IL-34 levels were independently associated with the KL grade (*B* = 0.273, 95% CI: 0.150–0.395; *P* < 0.001). SF IL-34 levels were significantly correlated with WOMAC scores (*r* = 0.265, 95% CI: 0.123–0.399; *P* < 0.001). The correlation between SF IL-34 levels and WOMAC scores was still significant after adjusting for confounding factors (*B* = 0.020, 95% CI: 0.001–0.038; *P* = 0.035) in OA patients.

**Conclusions:**

We found that IL-34 levels in SF were significantly associated with the radiographic and symptomatic severity of knee OA.

## 1. Introduction

Osteoarthritis (OA) is the most common degenerative disorder of the joint, which is characterized by articular cartilage destruction, subchondral sclerosis, and synovitis [1]. Knee OA is the most prevalent joint disease causing limited mobility and diminished quality of life in the elderly [2].

Biochemical markers show promise in the evaluation of the severity of the disease in addition to monitoring the efficacy and safety of disease-modifying OA drugs [3]. The identification of reliable biochemical markers largely depends on understanding the biological or pathological mechanisms of OA. Although the etiology of OA is unclear, accumulating evidence has underlined that inflammation plays a key role in OA pathogenesis. Therefore, proinflammatory cytokines secreted from infiltrating inflammatory cells could be used as biochemical markers of OA.

Interleukin-34 (IL-34) is a novel cytokine identified by Lin et al. in 2008 [4]. The human IL-34 protein is composed of 222 amino acids and has a molecular mass of 39 kDa. IL-34 binds to a macrophage colony-stimulating factor (M-CSF) receptor and acts as a key regulator of the differentiation, proliferation, and survival of cells from the mononuclear phagocyte lineage [5, 6]. Previous studies have revealed that IL-34 is expressed in synovium, and the increased IL-34 levels in serum and synovial fluid (SF) are associated with synovitis severity and disease progression in rheumatoid arthritis (RA) patients [7–9]. However, the relationship between serum and SF levels of IL-34 and OA has never been fully illustrated. Therefore, we aimed to detect IL-34 levels in the serum and SF of OA patients and to investigate their potential correlation with the severity and functional status of knee OA.

## 2. Materials and Methods

### 2.1. Study Population

From August 2015 to May 2017, a total of 182 knee OA patients from Renji Hospital were invited to enroll in our study. The diagnosis of knee OA was determined according to the clinical and radiological criteria of the American College of Rheumatology [10]. Sixty-nine age- and sex-matched volunteers undergoing routine physical examination in Renji Hospital were recruited as healthy controls during the same period. The exclusion criteria were as follows: previous knee injury or joint infection, secondary posttraumatic OA, systemic inflammatory or autoimmune disorders, known malignant tumor, end-stage renal or hepatic disease, diabetes, and histories of corticosteroid medication. The research protocol was approved by the ethics committee of Renji Hospital. Written informed consent was obtained from all participants before initiating the study.

### 2.2. Sample Collection and Laboratory Tests

Blood samples from all patients and controls were collected after overnight fast in plain tubes containing a separation gel. SF samples were obtained from the affected knee of OA patients before the treatment of hyaluronic acid injection or during the arthroscopy or surgery. No SF samples were collected from the controls for ethical concerns. Samples were collected into sodium heparin Vacutainer tubes (Becton Dickinson). Blood and SF samples were centrifuged and stored at −80°C until investigation. High-sensitivity CRP (hs-CRP) levels were measured in a Tecan Freedom EVOlyzer Automatic Biochemical Analyzer System (Tecan, Switzerland). IL-34 levels in serum and SF were determined using the commercial enzyme-linked immunosorbent assay (ELISA) kit (R&D Systems, Minneapolis, MN, USA) according to the manufacturer's instructions. All the samples were synchronously and randomly detected by different ELISA kits. All the results of different kits were distributed similarly. According to the manufacturer, the intra-assay CV was 1.8% to 7.3% and the interassay CV was 4.1% to 6.0%. All measurements were taken in duplicate for each sample, and the results were averaged.

### 2.3. Radiographic Definitions

All patients underwent weight-bearing anteroposterior radiographs of the affected knee. The Kellgren-Lawrence (KL) grading system was used for classifying radiographic signs: grade 1, questionable narrowing of joint space and possible osteophytic lipping; grade 2, definite osteophytes and possible narrowing of joint space; grade3, moderate multiple osteophytes, definite narrowing of joint space, some sclerosis, and possible deformity of bone contour; and grade 4, large osteophytes, marked narrowing of joint space, severe sclerosis, and definite deformity of bone contour [11]. Two specialist surgeons analyzed the radiographic parameters.

### 2.4. Symptomatic Definitions

The symptomatic disease severity was evaluated according to Western Ontario McMaster University Osteoarthritis Index (WOMAC) [12]. WOMAC is a questionnaire containing 24 items in three domains: 5 items for pain, 2 for stiffness, and 17 for functional limitation. Scores are on a scale of 0 to 4, with four being the highest score. Higher WOMAC scores indicate greater symptom severity.

### 2.5. Statistical Analysis

Continuous data were presented as mean and standard deviation. Categorical variables were summarized as percentages. Normality assessment was performed with the Shapiro-Wilk test. A comparison of two independent groups was performed with the unpaired *t*-test, Mann–Whitney *U* (exact) test, or chi-squared test when appropriate. Differences among groups were analyzed by one-way analysis of variance (ANOVA) followed by Tukey post hoc analysis. As the KL grade is an ordinal result, linear regression in univariant and multivariant ways (after adjusting for age, sex, BMI, and WOMAC scores) was performed to evaluate the association between IL-34 levels and KL grades. Coefficient correlation between IL-34 levels and WOMAC scores was assessed by Spearman rank correlation. Possible independent relationship between variables (e.g., age, sex, BMI, IL-34 levels, and KL grade) and WOMAC scores was determined by multivariate linear regression. If the dependent variables in linear regression were not normally distributed, then logarithmic (log) transformed values were performed. Statistical analyses of the variables were conducted with SPSS 22.0 for Windows (SPSS Inc., Chicago, Illinois, USA). *P* value less than 0.05 (two-tailed) was accepted as statistically significant.

## 3. Results

### 3.1. Baseline Clinical Characteristics

The baseline clinical characteristics of the subjects were shown in Table 1. No significant differences were observed in age, gender, and body mass index (BMI) between OA patients and controls (*P* > 0.05). In the OA group, there were also no significant differences in baseline clinical characteristics among patients with different KL grades (*P* > 0.05).

### 3.2. Serum and SF IL-34 Levels

As shown in Table 1, there was no significant difference in serum IL-34 levels between OA patients and controls. In OA patients, there was also no significant difference in serum IL-34 levels among patients with different KL grades (*P* > 0.05). In OA patients, IL-34 levels in SF were significantly higher than those in paired serum samples (*P* < 0.01). And SF IL-34 levels significantly increased with the increment of the KL grade (*P* < 0.01).

### 3.3. Correlation of IL-34 Levels with KL Grades

As shown in Table 2, the KL grade was not associated with serum IL-34 levels in univariant (*B* = 0.034, 95% CI: −0.072–0.140; *P* = 0.528) and multivariant ways (*B* = 0.019, 95% CI: −0.106–0.144; *P* = 0.306) in OA patients. However, as shown in Table 3, multivariate linear regression showed that the KL grade was independently associated with SF IL-34 levels (*B* = 0.273, 95% CI 0.150–0.395; *P* < 0.001).

### 3.4. Correlation of IL-34 Levels with WOMAC Scores

**Figure 1:**
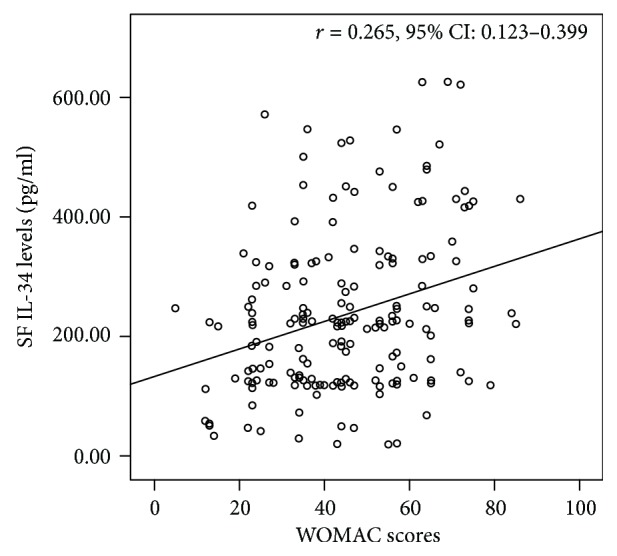
Correlations of SF IL-34 levels with WOMAC scores. SF = synovial fluid; IL = interleukin; WOMAC scores = Western Ontario and McMaster Universities Arthritis Index scores.

In OA patients, SF IL-34 levels were significantly associated with WOMAC scores (*r* = 0.265, 95% CI: 0.123–0.399; *P* < 0.001; Figure 1). As shown in Table 4, the KL grade was not associated with serum IL-34 levels in univariant (*B* = 0.034, 95% CI: −0.072–0.140; *P* = 0.528) and multivariant ways (*B* = 0.019, 95% CI: −0.106–0.144; *P* = 0.306). Univariate linear regression showed that the SF IL-34 level was associated with WOMAC scores (*B* = 0.038, 95% CI: 0.020–0.056; *P* < 0.001). Multivariate linear regression analysis also showed that this correlation was still significant after adjusting for confounding factors (*B* = 0.020, 95% CI: 0.001–0.038; *P* = 0.035). Besides, the KL grade was also independently associated with WOMAC scores (*B* = 0.212, 95% CI: 0.130–0.293; *P* < 0.001).

## 4. Discussion

Considerable attention has been paid recently to the identification of biomarkers for OA. In the present study, we found that IL-34 levels in SF were significantly higher than those in paired serum samples in OA patients. Moreover, SF IL-34 levels were independently associated with the symptomatic and radiographic severity of the disease. These results indicated that IL-34 might play a significant role in the synovial inflammation of OA.

Biochemical biomarkers with sensitivity and reliability can facilitate early diagnosis of joint destruction, disease prognosis, and progression monitoring. Although historically OA was considered a degenerative disorder, recent studies have found that inflammation might be the primary trigger of the OA process, which results in cartilage damage [13]. Monocytes and macrophages are the major cells involved in the pathogenesis of inflammatory arthritis [14]. As one of the key regulators of monocyte/macrophage survival, IL-34 can be produced by fibroblast-like synoviocytes and released into circulation and SF [8]. Previous studies have revealed that IL-34 levels were elevated in the serum and SF of RA patients. We found in the present study that no significant differences were found in serum IL-34 levels between OA patients and healthy controls. These results are consistent with the theory that OA is a low-grade local inflammation and thus inherently lacks the large-scale systemic response of RA [15]. We also revealed that IL-34 levels in SF were significantly higher than those in paired serum samples in OA patients. These results indicated that IL-34 had a limited function to transfer across the synovial membrane due to its molecular weight or complex structure, and IL-34 levels in SF might reflect the intra-articular expression of this proinflammatory mediator.

Close monitoring of radiographic progression may facilitate the design of proper therapies. We found that the KL grade was associated with SF levels but not with serum SF levels. After adjusting for confounding factors, the KL grade was still independently associated with SF IL-34 levels. These results revealed the association between SF IL-34 levels and the radiographic severity of OA. As a macrophage colony-stimulating factor, IL-34 can stimulate the viability of monocytes and colony formation of macrophages [4]. The inflammatory monocytes can infiltrate the joints into osteoclasts that cause osteoporosis [16]. Moreover, IL-34 can also induce both growth and proliferation in osteoclast progenitors and play a key role in the receptor activator of nuclear factor kappa-B ligand-induced osteoclastogenesis [17, 18]. These mechanisms may partly explain the results we observed in the present study.

We also found that SF IL-34 levels were significantly associated with self-reported knee pain after adjustment for confounding factors. Inflammatory cytokines can activate innervating nociceptors and increase excitability of sensory neurons [19]. Therefore, intra-articular inflammation may lead to the generation and maintenance of pain, which is a major cause of disability and functional decline. Several inflammatory cytokines, including IL-6, IL-8, and tumor necrosis factor-*α* have been reported to correlate with OA-related pain [20, 21]. Furthermore, pain can be related to the progression of structural changes and cartilage degradation of OA [21], as we have showed in this study that the KL grade was also independently associated with WOMAC scores.

Our study has several limitations. First, we were limited by the trial design. This cross-sectional study showed association but not causation. In addition, our sample size remains small. These findings will require further replication, validation, and qualification in large, longitudinal population cohorts. Second, although SF biomarkers provide a more proximal indicator of the disease state than serum biomarkers, they do not provide definitive indications of their tissue of origin. Third, as patients with synovial effusion can be a different pattern of knee OA, analyzing these patients would be interesting. However, we did not have data concerning these patients. Fourth, the control enrolled in this study underwent a clinical examination but no X-ray explanation was performed. Therefore, we cannot evaluate if control patients had radiographic knee OA, which might induce some bias.

In conclusion, we showed for the first time that IL-34 levels in SF were significantly associated with the radiographic and symptomatic severity of knee OA.

## Figures and Tables

**Table 1:** Baseline clinical characteristics and IL-34 levels.

	Controls (*n* = 69)	OA patients total (*n* = 182)	KL grade 2 (*n* = 77)	KL grade 3 (*n* = 58)	KL grade 4 (*n* = 47)
Age (years)	64 (56–74)	67 (59–73)	66 (58–74)	68 (61–72)	66 (61–72)
Female, *n* (%)	44 (68.75%)	122 (67.03%)	47 (71.21%)	42 (61.76%)	33 (70.21%)
BMI (kg/mm^2^)	22.96 ± 2.29	23.30 ± 2.11	23.21 ± 2.08	23.25 ± 2.21	23.50 ± 2.07
WOMAC scores		44 (33–57)	34 (23–45)	46 (36–57)	57 (44–65)
Serum IL-34 levels (pg/ml)	129.87 (88.66–214.49)	121.35 (86.84–177.70)	107.50 (80.19–195.32)	128.33 (98.06–196.94)	121.25 (91.01–172.32)
SF IL-34 levels (pg/ml)		222.55 (126.68–319.75)	154.10 (117.71–237.07)	226.44 (147.23–329.63)	256.18 (212.09–432.08)

All values are expressed as the percentages, median (interquartile range), or mean ± SD. IL-34: interleukin-34; OA: osteoarthritis; KL grade: Kellgren-Lawrence grade; BMI: body mass index; WOMAC: Western Ontario McMaster University Osteoarthritis Index; SF: synovial fluid.

**Table 2:** Linear regression for the association between variables and serum IL-34 levels.

Variables	Univariant		Multivariant	
	*B* (95% CI)	*P* value	*B* (95% CI)	*P* value
Age	−0.004 (−0.011–0.003)	0.295	−0.013 (−0.026–0.001)	0.064
Sex	−0.042 (−0.192–0.108)	0.583	−0.099 (−0.291–0.093)	0.311
BMI	0.011 (−0.022–0.044)	0.505	0.052 (−0.004–0.108)	0.066
SF IL-34 levels	<0.001 (−0.001–0.001)	0.411	<0.001 (−0.001–0.001)	0.684
KL grade	0.034 (−0.072–0.140)	0.528	0.019 (−0.106–0.144)	0.306
WOMAC scores	0.002 (−0.003–0.007)	0.425	0.002 (−0.004–0.008)	0.529

CI = confidence interval. Other abbreviations are as in Table 1.

**Table 3:** Linear regression for the association between variables and SF IL-34 levels.

Variables	Univariant		Multivariant	
	*B* (95% CI)	*P* value	*B* (95% CI)	*P* value
Age	−0.008 (−0.018–0.002)	0.117	−0.010 (−0.023–0.002)	0.112
Sex	−0.050 (−0.256–0.155)	0.631	−0.080 (−0.279–0.119)	0.427
BMI	−0.022 (−0.068–0.024)	0.341	−0.003 (−0.062–0.055)	0.910
Serum IL-34 levels	<0.001 (−0.001–0.001)	0.667	<0.001 (−0.001–0.001)	0.480
KL grade	0.322 (0.212–0.432)	<0.001	0.273 (0.150–0.395)	<0.001
WOMAC scores	0.010 (0.005–0.016)	<0.001	0.005 (0.001–0.011)	0.034

All abbreviations are as in Tables 1 and 2.

**Table 4:** Linear regression for the association between variables and WOMAC scores.

Variables	Univariant		Multivariant	
	*B* (95% CI)	*P* value	*B* (95% CI)	*P* value
Age	0.004 (−0.003–0.010)	0.305	0.002 (−0.007–0.011)	0.672
Sex	−0.024 (−0.165–0.118)	0.742	0.045 (−0.009–0.180)	0.510
BMI	0.023 (−0.009–0.054)	0.157	0.016 (−0.024–0.056)	0.429
Serum IL-34 levels	<0.001 (−0.001–0.001)	0.683	<0.001 (−0.001–0.001)	0.761
SF IL-34 levels	0.038 (0.020–0.056)	<0.001	0.020 (0.001–0.038)	0.035
KL grade	0.244 (0.170–0.318)	<0.001	0.212 (0.130–0.293)	<0.001

All abbreviations are as in Tables 1 and 2.

## Data Availability

The SPSS Statistics Data Document.sav data used to support the findings of this study are available from the corresponding author (Email: shlizhanchun@21cn.com) upon request.

## References

[1] Pulsatelli L., Addimanda O., Brusi V., Pavloska B., Meliconi R. (2013). New findings in osteoarthritis pathogenesis: therapeutic implications. *Therapeutic Advances in Chronic Disease*.

[2] Felson D. T., Zhang Y., Hannan M. T. (1995). The incidence and natural history of knee osteoarthritis in the elderly. The Framingham Osteoarthritis Study. *Arthritis & Rheumatism*.

[3] Mabey T., Honsawek S. (2015). Cytokines as biochemical markers for knee osteoarthritis. *World Journal of Orthopedics*.

[4] Lin H., Lee E., Hestir K. (2008). Discovery of a cytokine and its receptor by functional screening of the extracellular proteome. *Science*.

[5] Chihara T., Suzu S., Hassan R. (2010). IL-34 and M-CSF share the receptor Fms but are not identical in biological activity and signal activation. *Cell Death and Differentiation*.

[6] Wei S., Nandi S., Chitu V. (2010). Functional overlap but differential expression of CSF-1 and IL-34 in their CSF-1 receptor-mediated regulation of myeloid cells. *Journal of Leukocyte Biology*.

[7] Chemel M., Le Goff B., Brion R. (2012). Interleukin 34 expression is associated with synovitis severity in rheumatoid arthritis patients. *Annals of the Rheumatic Diseases*.

[8] Tian Y., Shen H., Xia L., Lu J. (2013). Elevated serum and synovial fluid levels of interleukin-34 in rheumatoid arthritis: possible association with disease progression via interleukin-17 production. *Journal of Interferon & Cytokine Research*.

[9] Moon S. J., Hong Y. S., Ju J. H., Kwok S. K., Park S. H., Min J. K. (2013). Increased levels of interleukin 34 in serum and synovial fluid are associated with rheumatoid factor and anticyclic citrullinated peptide antibody titers in patients with rheumatoid arthritis. *The Journal of Rheumatology*.

[10] Altman R., Asch E., Bloch D. (1986). Development of criteria for the classification and reporting of osteoarthritis. Classification of osteoarthritis of the knee. Diagnostic and Therapeutic Criteria Committee of the American Rheumatism Association. *Arthritis & Rheumatism*.

[11] Kellgren J. H., Lawrence J. S. (1957). Radiological assessment of osteo-arthrosis. *Annals of the Rheumatic Diseases*.

[12] Bellamy N., Buchanan W. W., Goldsmith C. H., Campbell J., Stitt L. W. (1988). Validation study of WOMAC: a health status instrument for measuring clinically important patient relevant outcomes to antirheumatic drug therapy in patients with osteoarthritis of the hip or knee. *The Journal of Rheumatology*.

[13] Nguyen L. T., Sharma A. R., Chakraborty C., Saibaba B., Ahn M. E., Lee S. S. (2017). Review of prospects of biological fluid biomarkers in osteoarthritis. *International Journal of Molecular Sciences*.

[14] Darrieutort-Laffite C., Boutet M. A., Chatelais M. (2014). IL-1*β* and TNF*α* promote monocyte viability through the induction of GM-CSF expression by rheumatoid arthritis synovial fibroblasts. *Mediators of Inflammation*.

[15] Stürmer T., Brenner H., Koenig W., Günther K. P. (2004). Severity and extent of osteoarthritis and low grade systemic inflammation as assessed by high sensitivity C reactive protein. *Annals of the Rheumatic Diseases*.

[16] Hwang S. J., Choi B., Kang S. S. (2012). Interleukin-34 produced by human fibroblast-like synovial cells in rheumatoid arthritis supports osteoclastogenesis. *Arthritis Research & Therapy*.

[17] Chen Z., Buki K., Vääräniemi J., Gu G., Väänänen H. K. (2011). The critical role of IL-34 in osteoclastogenesis. *PLoS One*.

[18] Baud'huin M., Renault R., Charrier C. (2010). Interleukin-34 is expressed by giant cell tumours of bone and plays a key role in RANKL-induced osteoclastogenesis. *The Journal of Pathology*.

[19] Miller R. E., Miller R. J., Malfait A. M. (2014). Osteoarthritis joint pain: the cytokine connection. *Cytokine*.

[20] Kapoor M., Martel-Pelletier J., Lajeunesse D., Pelletier J. P., Fahmi H. (2011). Role of proinflammatory cytokines in the pathophysiology of osteoarthritis. *Nature Reviews Rheumatology*.

[21] Leung Y. Y., Huebner J. L., Haaland B., Wong S. B. S., Kraus V. B. (2017). Synovial fluid pro-inflammatory profile differs according to the characteristics of knee pain. *Osteoarthritis and Cartilage*.

